# Supplementary material for: A Likelihood Approach for Real-Time Calibration of Stochastic Compartmental Epidemic Models
Source: PLoS Comput Biol. 2017 Jan 17;13(1):e1005257. doi: 10.1371/journal.pcbi.1005257 (PMC5240920; doi:10.1371/journal.pcbi.1005257)
Supplement: S1 File — (TAR.GZ) [file pcbi.1005257.s014.tar.gz › HSPH_Online-SI-Revision/PF08/n10-mild/n10-mild_PF2_table8.pdf]

1	newly 3 weeks spec	60.	160.49	{141., 179.}	167.483	0.425191	newly 3 weeks cumul	239.	526.83	{493., 558.}	120.431	0.342672	newly 5 weeks spec	59.	130.68	{114., 147.}	121.492	0.342917	newly 5 weeks cumul	350.	801.2	{751., 847.}	128.914	0.359122	newly 42 weeks cumul	920.	1727.5	{1572., 1893.}	87.7717	0.272476
2	newly 3 weeks spec	35.	149.61	{120., 190.}	327.457	0.623788	newly 3 weeks cumul	125.	521.94	{450., 625.}	317.552	0.616792	newly 5 weeks spec	13.	103.49	{78., 135.}	696.077	0.8899	newly 5 weeks cumul	172.	751.12	{640., 908.}	336.698	0.635408	newly 47 weeks cumul	189.	1240.5	{982., 1617.}	556.349	0.808474
3	newly 3 weeks spec	49.	199.61	{166., 232.}	307.367	0.60646	newly 3 weeks cumul	214.	708.56	{650., 769.}	231.103	0.518279	newly 5 weeks spec	21.	135.75	{109., 161.}	546.429	0.804121	newly 5 weeks cumul	265.	1011.04	{914., 1103.}	281.525	0.579344	newly 50 weeks cumul	281.	1582.39	{1388., 1755.}	463.128	0.746251
4	newly 3 weeks spec	42.	155.63	{117., 197.}	270.548	0.559635	newly 3 weeks cumul	187.	538.4	{438., 650.}	187.914	0.453321	newly 5 weeks spec	18.	113.02	{76., 155.}	527.889	0.782825	newly 5 weeks cumul	235.	788.18	{619., 981.}	235.396	0.518028	newly 45 weeks cumul	289.	1393.08	{1033., 2009.}	382.035	0.669053
5	newly 3 weeks spec	82.	19.34	{14., 26.}	76.4146	0.64209	newly 3 weeks cumul	270.	68.51	{61., 76.}	74.6259	0.597044	newly 5 weeks spec	96.	14.33	{9., 21.}	85.0729	0.854688	newly 5 weeks cumul	451.	99.78	{90., 110.}	77.8758	0.656516	newly 25 weeks cumul	1188.	188.55	{149., 236.}	84.1288	0.805916
6	newly 3 weeks spec	58.	269.78	{218., 313.}	365.138	0.663241	newly 3 weeks cumul	223.	885.1	{784., 971.}	296.906	0.596321	newly 5 weeks spec	18.	215.79	{162., 258.}	1098.83	1.07062	newly 5 weeks cumul	288.	1346.95	{1136., 1515.}	367.691	0.666616	newly 51 weeks cumul	298.	2635.31	{1932., 2949.}	784.332	0.938957
7	newly 3 weeks spec	72.	174.94	{147., 202.}	142.972	0.381751	newly 3 weeks cumul	231.	590.26	{537., 636.}	155.524	0.405364	newly 5 weeks spec	20.	137.71	{112., 168.}	588.55	0.831141	newly 5 weeks cumul	292.	883.66	{802., 965.}	202.623	0.47845	newly 47 weeks cumul	336.	1714.08	{1447., 1904.}	410.143	0.702467
8	newly 3 weeks spec	137.	162.97	{142., 180.}	19.146	0.0742443	newly 3 weeks cumul	446.	539.35	{502., 575.}	20.9305	0.0820343	newly 5 weeks spec	94.	129.7	{113., 149.}	38.0638	0.137131	newly 5 weeks cumul	661.	814.45	{757., 863.}	23.2148	0.0901344	newly 42 weeks cumul	1278.	1706.35	{1535., 1871.}	33.5172	0.124292
9	newly 3 weeks spec	36.	188.42	{151., 229.}	423.389	0.71275	newly 3 weeks cumul	178.	644.12	{545., 728.}	261.865	0.555262	newly 5 weeks spec	6.	139.18	{106., 174.}	2219.67	1.3574	newly 5 weeks cumul	197.	944.7	{768., 1078.}	379.543	0.676768	newly 46 weeks cumul	197.	1651.56	{1234., 2101.}	738.355	0.914865
10	newly 3 weeks spec	48.	153.14	{121., 204.}	219.042	0.495155	newly 3 weeks cumul	183.	507.57	{428., 658.}	177.361	0.436988	newly 5 weeks spec	54.	122.69	{86., 167.}	127.204	0.34478	newly 5 weeks cumul	294.	765.86	{627., 994.}	160.497	0.409091	newly 43 weeks cumul	610.	1616.79	{1254., 2108.}	165.048	0.414101
11	newly 3 weeks spec	38.	217.64	{172., 273.}	472.737	0.751131	newly 3 weeks cumul	143.	691.83	{553., 843.}	383.797	0.679531	newly 5 weeks spec	2.	188.	{141., 241.}	9300.	1.96391	newly 5 weeks cumul	158.	1081.82	{877., 1310.}	584.696	0.829662	newly 48 weeks cumul	209.	2520.24	{1913., 3163.}	1105.86	1.07226
12	newly 3 weeks spec	4.	220.93	{195., 240.}	5423.25	1.74075	newly 3 weeks cumul	21.	818.5	{779., 861.}	3797.62	1.59046	newly 5 weeks spec	0.1	138.84	{115., 162.}	138740.	3.13894	newly 5 weeks cumul	21.	1131.44	{1067., 1207.}	5287.81	1.73094	newly 50 weeks cumul	21.	1597.79	{1457., 1734.}	7508.52	1.88039
13	newly 3 weeks spec	14.	154.54	{126., 178.}	1003.86	1.03881	newly 3 weeks cumul	103.	525.16	{459., 582.}	409.864	0.704445	newly 5 weeks spec	15.	117.69	{95., 135.}	684.6	0.888436	newly 5 weeks cumul	131.	777.87	{669., 863.}	493.794	0.770319	newly 42 weeks cumul	149.	1474.19	{1226., 1672.}	889.389	0.990178
14	newly 3 weeks spec	78.	163.61	{128., 190.}	109.756	0.316832	newly 3 weeks cumul	298.	549.87	{444., 618.}	84.5201	0.262888	newly 5 weeks spec	48.	127.73	{97., 152.}	166.104	0.418778	newly 5 weeks cumul	412.	824.29	{643., 924.}	100.07	0.29766	newly 43 weeks cumul	587.	1633.37	{1231., 1897.}	178.257	0.438236
15	newly 3 weeks spec	142.	214.03	{190., 237.}	50.7254	0.175968	newly 3 weeks cumul	509.	693.6	{643., 739.}	36.2672	0.133328	newly 5 weeks spec	97.	176.32	{143., 200.}	81.7732	0.256254	newly 5 weeks cumul	755.	1065.3	{985., 1130.}	41.0993	0.148443	newly 45 weeks cumul	1453.	2325.88	{2066., 2546.}	60.0743	0.202039
16	newly 3 weeks spec	54.	188.15	{154., 223.}	248.426	0.536386	newly 3 weeks cumul	234.	632.79	{547., 713.}	170.423	0.428571	newly 5 weeks spec	35.	145.01	{109., 187.}	314.314	0.609116	newly 5 weeks cumul	327.	944.29	{827., 1092.}	188.774	0.456752	newly 46 weeks cumul	351.	1765.95	{1450., 2298.}	403.12	0.693294
17	newly 3 weeks spec	90.	180.3	{153., 202.}	100.333	0.299469	newly 3 weeks cumul	301.	592.62	{548., 621.}	96.8837	0.293751	newly 5 weeks spec	86.	148.39	{127., 169.}	72.5465	0.23432	newly 5 weeks cumul	488.	904.99	{845., 946.}	85.4488	0.267823	newly 43 weeks cumul	2262.	1992.93	{1838., 2177.}	11.8952	0.0557997
18	newly 3 weeks spec	41.	184.56	{164., 205.}	350.146	0.650653	newly 3 weeks cumul	174.	605.61	{576., 642.}	248.052	0.540008	newly 5 weeks spec	45.	149.13	{128., 170.}	231.4	0.515893	newly 5 weeks cumul	264.	922.87	{872., 981.}	249.572	0.541748	newly 43 weeks cumul	314.	2000.23	{1828., 2179.}	537.016	0.800637
19	newly 3 weeks spec	42.	154.07	{122., 180.}	266.833	0.559073	newly 3 weeks cumul	145.	526.04	{453., 593.}	262.786	0.556903	newly 5 weeks spec	27.	117.51	{88., 148.}	335.222	0.629635	newly 5 weeks cumul	208.	779.72	{658., 892.}	274.865	0.570427	newly 46 weeks cumul	266.	1436.6	{1118., 1735.}	440.075	0.725095
20	newly 3 weeks spec	94.	181.89	{125., 229.}	93.5	0.277127	newly 3 weeks cumul	348.	617.17	{462., 727.}	77.3477	0.242716	newly 5 weeks spec	48.	135.01	{87., 175.}	181.271	0.433341	newly 5 weeks cumul	453.	911.28	{653., 1096.}	101.166	0.295627	newly 47 weeks cumul	551.	1648.9	{1054., 2127.}	199.256	0.460004
21	newly 3 weeks spec	33.	165.73	{127., 206.}	402.212	0.692907	newly 3 weeks cumul	145.	569.24	{466., 691.}	292.579	0.589318	newly 5 weeks spec	16.	123.82	{85., 169.}	673.875	0.873131	newly 5 weeks cumul	184.	836.74	{675., 1008.}	354.75	0.651743	newly 47 weeks cumul	205.	1459.46	{1069., 1841.}	611.932	0.840501
22	newly 3 weeks spec	79.	169.46	{151., 192.}	114.506	0.329175	newly 3 weeks cumul	247.	559.03	{518., 595.}	126.328	0.353846	newly 5 weeks spec	60.	137.61	{119., 157.}	129.35	0.357809	newly 5 weeks cumul	392.	848.82	{782., 910.}	116.536	0.334611	newly 43 weeks cumul	671.	1829.15	{1657., 1997.}	172.601	0.433913
23	newly 3 weeks spec	1.	141.98	{119., 164.}	14098.	2.14807	newly 3 weeks cumul	20.	511.	{467., 555.}	2455.	1.40567	newly 5 weeks spec	0.1	96.89	{76., 117.}	96790.	2.97844	newly 5 weeks cumul	20.	727.72	{651., 795.}	3538.6	1.55868	newly 48 weeks cumul	20.	1156.73	{1015., 1268.}	5683.65	1.75756
24	newly 3 weeks spec	31.	154.46	{104., 210.}	398.258	0.682894	newly 3 weeks cumul	126.	518.52	{380., 688.}	311.524	0.604241	newly 5 weeks spec	21.	125.57	{79., 178.}	497.952	0.757046	newly 5 weeks cumul	167.	785.41	{559., 1066.}	370.305	0.660444	newly 44 weeks cumul	190.	1621.88	{1000., 2393.}	753.621	0.908278
25	newly 3 weeks spec	26.	306.97	{230., 367.}	1080.65	1.06504	newly 3 weeks cumul	124.	1001.55	{790., 1161.}	707.702	0.902872	newly 5 weeks spec	3.	241.25	{149., 307.}	7941.67	1.89101	newly 5 weeks cumul	138.	1516.06	{1150., 1775.}	998.594	1.0347	newly 51 weeks cumul	140.	2911.19	{1841., 3695.}	1979.42	1.30261
26	newly 3 weeks spec	39.	141.87	{92., 192.}	263.769	0.543458	newly 3 weeks cumul	98.	473.12	{318., 619.}	382.776	0.670433	newly 5 weeks spec	10.	108.95	{66., 153.}	989.5	1.01556	newly 5 weeks cumul	122.	705.25	{462., 931.}	478.074	0.747162	newly 42 weeks cumul	155.	1419.05	{884., 1962.}	815.516	0.941387
27	newly 3 weeks spec	6.	150.77	{112., 205.}	2412.83	1.38961	newly 3 weeks cumul	37.	520.98	{414., 687.}	1308.05	1.14183	newly 5 weeks spec	1.	115.29	{78., 159.}	11429.	2.04646	newly 5 weeks cumul	40.	767.14	{587., 1024.}	1817.85	1.27495	newly 44 weeks cumul	46.	1397.24	{981., 1930.}	2937.48	1.46866
28	newly 3 weeks spec	58.	12.27	{7., 17.}	78.8448	0.700251	newly 3 weeks cumul	196.	40.86	{35., 48.}	79.1531	0.684615	newly 5 weeks spec	40.	8.66	{5., 12.}	78.35	0.68466	newly 5 weeks cumul	279.	59.24	{52., 69.}	78.767	0.675384	newly 22 weeks cumul	839.	112.12	{85., 138.}	86.6365	0.881786
29	newly 3 weeks spec	51.	136.65	{94., 181.}	167.941	0.415295	newly 3 weeks cumul	209.	474.4	{359., 587.}	126.986	0.347941	newly 5 weeks spec	29.	104.38	{64., 149.}	259.931	0.537028	newly 5 weeks cumul	281.	699.	{508., 866.}	148.754	0.38606	newly 45 weeks cumul	388.	1282.8	{808., 1710.}	230.619	0.499887
30	newly 3 weeks spec	44.	143.21	{110., 181.}	225.477	0.504519	newly 3 weeks cumul	129.	484.19	{400., 581.}	275.341	0.569015	newly 5 weeks spec	32.	109.35	{78., 147.}	241.719	0.521299	newly 5 weeks cumul	194.	718.29	{571., 868.}	270.253	0.562119	newly 44 weeks cumul	268.	1370.89	{976., 1886.}	411.526	0.694251
31	newly 3 weeks spec	39.	271.66	{250., 294.}	596.564	0.842038	newly 3 weeks cumul	120.	862.59	{811., 922.}	618.825	0.856095	newly 5 weeks spec	8.	230.68	{207., 255.}	2783.5	1.4586	newly 5 weeks cumul	150.	1344.67	{1275., 1424.}	796.447	0.952115	newly 51 weeks cumul	165.	3037.58	{2884., 3181.}	1740.96	1.26469
32	newly 3 weeks spec	110.	16.81	{11., 23.}	84.7182	0.833009	newly 3 weeks cumul	385.	58.75	{51., 68.}	84.7403	0.819433	newly 5 weeks spec	87.	12.64	{7., 18.}	85.4713	0.864372	newly 5 weeks cumul	596.	86.59	{73., 99.}	85.4715	0.840934	newly 24 weeks cumul	1460.	162.17	{128., 197.}	88.8925	0.961117
33	newly 3 weeks spec	26.	155.53	{121., 194.}	498.192	0.768746	newly 3 weeks cumul	122.	544.88	{441., 648.}	346.623	0.644859	newly 5 weeks spec	8.	112.73	{81., 147.}	1309.13	1.13629	newly 5 weeks cumul	152.	790.44	{634., 959.}	420.026	0.710135	newly 45 weeks cumul	158.	1366.94	{1035., 1798.}	765.152	0.927022
34	newly 3 weeks spec	95.	195.68	{152., 230.}	106.274	0.309128	newly 3 weeks cumul	318.	638.9	{528., 713.}	100.912	0.299645	newly 5 weeks spec	73.	157.81	{123., 189.}	116.726	0.329699	newly 5 weeks cumul	481.	972.3	{789., 1097.}	102.154	0.301867	newly 45 weeks cumul	672.	2044.88	{1519., 2409.}	204.298	0.475658
35	newly 3 weeks spec	44.	142.64	{110., 187.}	224.182	0.502475	newly 3 weeks cumul	154.	498.26	{411., 616.}	223.545	0.503905	newly 5 weeks spec	28.	104.21	{76., 133.}	272.179	0.557963	newly 5 weeks cumul	217.	725.31	{596., 915.}	234.244	0.517236	newly 45 weeks cumul	280.	1267.28	{968., 1740.}	352.6	0.644374
36	newly 3 weeks spec	9.	179.3	{146., 217.}	1892.22	1.29334	newly 3 weeks cumul	71.	615.67	{530., 719.}	767.141	0.934564	newly 5 weeks spec	1.	133.19	{99., 176.}	13219.	2.1145	newly 5 weeks cumul	72.	905.25	{756., 1081.}	1157.29	1.0949	newly 47 weeks cumul	75.	1636.37	{1220., 2133.}	2081.83	1.32838
37	newly 3 weeks spec	26.	204.02	{164., 246.}	684.692	0.889228	newly 3 weeks cumul	60.	663.66	{579., 752.}	1006.1	1.04131	newly 5 weeks spec	12.	164.82	{121., 218.}	1273.5	1.12623	newly 5 weeks cumul	86.	1008.3	{848., 1180.}	1072.44	1.06526	newly 50 weeks cumul	182.	2038.46	{1461., 2807.}	1020.03	1.03297
38	newly 3 weeks spec	105.	186.87	{144., 234.}	77.9714	0.243012	newly 3 weeks cumul	339.	620.18	{498., 738.}	82.944	0.256976	newly 5 weeks spec	72.	144.23	{108., 187.}	100.625	0.291657	newly 5 weeks cumul	490.	930.37	{738., 1119.}	89.8714	0.272113	newly 46 weeks cumul	831.	1796.71	{1275., 2353.}	116.211	0.322779
39	newly 3 weeks spec	5.	195.25	{170., 216.}	3805.	1.58971	newly 3 weeks cumul	20.	646.06	{595., 685.}	3130.3	1.50862	newly 5 weeks spec	0.1	153.71	{127., 179.}	153610.	3.18285	newly 5 weeks cumul	20.	973.	{875., 1034.}	4765.	1.68621	newly 47 weeks cumul	20.	1922.14	{1466., 2139.}	9510.7	1.97903
40	newly 3 weeks spec	69.	9.94	{6., 15.}	85.5942	0.869098	newly 3 weeks cumul	225.	33.32	{27., 39.}	85.1911	0.833271	newly 5 weeks spec	51.	6.57	{3., 10.}	87.1176	0.933904	newly 5 weeks cumul	346.	47.9	{41., 56.}	86.1561	0.862187	newly 20 weeks cumul	809.	88.12	{68., 114.}	89.1075	0.971927
41	newly 3 weeks spec	0.1	249.85	{225., 271.}	249750.	3.39667	newly 3 weeks cumul	0.1	893.85	{857., 931.}	893750.	3.95103	newly 5 weeks spec	0.1	167.28	{149., 187.}	167180.	3.22146	newly 5 weeks cumul	0.1	1267.6	{1209., 1315.}	1.2675×10^{6}	4.10274	newly 51 weeks cumul	0.1	1935.81	{1804., 2043.}	1.93571×10^{6}	4.28636
42	newly 3 weeks spec	35.	156.06	{121., 196.}	345.886	0.641434	newly 3 weeks cumul	180.	519.94	{434., 613.}	188.856	0.455257	newly 5 weeks spec	44.	126.37	{96., 159.}	187.205	0.449117	newly 5 weeks cumul	253.	787.8	{652., 944.}	211.383	0.487389	newly 43 weeks cumul	319.	1636.53	{1276., 2076.}	413.019	0.700471
43	newly 3 weeks spec	0.1	265.48	{237., 295.}	265380.	3.4223	newly 3 weeks cumul	1.	911.59	{848., 964.}	91059.	2.95903	newly 5 weeks spec	0.1	201.16	{170., 235.}	201060.	3.30038	newly 5 weeks cumul	1.	1346.23	{1239., 1434.}	134523.	3.12812	newly 49 weeks cumul	1.	2361.41	{2063., 2660.}	236041.	3.37057
44	newly 3 weeks spec	90.	201.03	{159., 228.}	123.367	0.344952	newly 3 weeks cumul	310.	658.69	{533., 722.}	112.481	0.324666	newly 5 weeks spec	69.	160.23	{126., 190.}	132.217	0.36027	newly 5 weeks cumul	468.	996.47	{799., 1090.}	112.921	0.325259	newly 45 weeks cumul	766.	2109.95	{1558., 2437.}	175.45	0.434918
45	newly 3 weeks spec	7.	257.06	{226., 296.}	3572.29	1.56269	newly 3 weeks cumul	18.	950.88	{886., 1054.}	5182.67	1.72207	newly 5 weeks spec	3.	162.21	{138., 188.}	5307.	1.72947	newly 5 weeks cumul	26.	1320.69	{1214., 1494.}	4979.58	1.70475	newly 52 weeks cumul	26.	1895.08	{1719., 2294.}	7188.77	1.86043
46	newly 3 weeks spec	14.	226.19	{183., 276.}	1515.64	1.20222	newly 3 weeks cumul	72.	750.76	{641., 889.}	942.722	1.01466	newly 5 weeks spec	4.	174.95	{138., 227.}	4273.75	1.63242	newly 5 weeks cumul	77.	1125.03	{948., 1398.}	1361.08	1.16038	newly 49 weeks cumul	79.	2114.1	{1608., 2934.}	2576.08	1.41768
47	newly 3 weeks spec	62.	225.42	{173., 287.}	263.581	0.55135	newly 3 weeks cumul	217.	770.73	{629., 963.}	255.175	0.544545	newly 5 weeks spec	38.	161.02	{116., 217.}	323.737	0.613152	newly 5 weeks cumul	302.	1118.19	{873., 1421.}	270.262	0.561304	newly 49 weeks cumul	355.	1886.51	{1390., 2545.}	431.411	0.712838
48	newly 3 weeks spec	10.	178.18	{131., 245.}	1681.8	1.2381	newly 3 weeks cumul	99.	585.36	{482., 751.}	491.273	0.763807	newly 5 weeks spec	18.	136.17	{96., 198.}	656.5	0.860316	newly 5 weeks cumul	143.	877.02	{700., 1184.}	513.301	0.777825	newly 46 weeks cumul	178.	1766.18	{1272., 2560.}	892.236	0.977497
49	newly 3 weeks spec	135.	159.79	{128., 181.}	20.4519	0.0796952	newly 3 weeks cumul	399.	532.03	{438., 577.}	33.4812	0.123738	newly 5 weeks spec	100.	126.79	{98., 149.}	28.91	0.108155	newly 5 weeks cumul	612.	801.59	{654., 874.}	31.2369	0.116202	newly 42 weeks cumul	1281.	1650.44	{1274., 1865.}	30.8212	0.115208
50	newly 3 weeks spec	34.	150.99	{123., 189.}	344.088	0.642091	newly 3 weeks cumul	108.	508.6	{439., 603.}	370.926	0.669467	newly 5 weeks spec	11.	119.31	{91., 159.}	984.636	1.02596	newly 5 weeks cumul	144.	763.93	{652., 942.}	430.507	0.720602	newly 42 weeks cumul	204.	1542.1	{1243., 2039.}	655.931	0.870881
